# Genome-Wide Identification and Characterization of Small Peptides in Maize

**DOI:** 10.3389/fpls.2021.695439

**Published:** 2021-06-16

**Authors:** Yan Liang, Wanchao Zhu, Sijia Chen, Jia Qian, Lin Li

**Affiliations:** National Key Laboratory of Crop Genetic Improvement, Huazhong Agricultural University, Wuhan, China

**Keywords:** maize, small open reading frame, small peptide, Ribo-seq, mass spectrometry

## Abstract

Small peptides (sPeptides), <100 amino acids (aa) long, are encoded by small open reading frames (sORFs) often found in the 5′ and 3′ untranslated regions (or other parts) of mRNAs, in long non-coding RNAs, or transcripts from introns and intergenic regions; various sPeptides play important roles in multiple biological processes. In this study, we conducted a comprehensive study of maize (*Zea mays*) sPeptides using mRNA sequencing, ribosome profiling (Ribo-seq), and mass spectrometry (MS) on six tissues (each with at least two replicates). To identify maize sORFs and sPeptides from these data, we set up a robust bioinformatics pipeline and performed a genome-wide scan. This scan uncovered 9,388 sORFs encoding peptides of 2–100 aa. These sORFs showed distinct genomic features, such as different Kozak region sequences, higher specificity of translation, and high translational efficiency, compared with the canonical protein-coding genes. Furthermore, the MS data verified 2,695 sPeptides. These sPeptides perfectly discriminated all the tissues and were highly associated with their parental genes. Interestingly, the parental genes of sPeptides were significantly enriched in multiple functional gene ontology terms related to abiotic stress and development, suggesting the potential roles of sPeptides in the regulation of their parental genes. Overall, this study lays out the guidelines for genome-wide scans of sORFs and sPeptides in plants by integrating Ribo-seq and MS data and provides a more comprehensive resource of functional sPeptides in maize and gives a new perspective on the complex biological systems of plants.

## Introduction

Small peptides (sPeptides), which are defined as those peptides shorter than 100 amino acids (aa), represent a class of small molecules with important roles in various biological processes and are translated from small open reading frames (sORFs) shorter than 300 nucleotides (Wang S. et al., 2020). sORFs are widely distributed in the genome and are likely to be located at 5′ and 3′ ends in the untranslated regions of mRNAs [upstream ORFs (uORFs) and downstream ORFs (dORFs)], in the internal regions of annotated ORFs of mRNAs (mORFs) but in a different reading frame, in the short isoforms in spliced mRNAs, and in RNAs produced by transcribed loci in the introns or intergenic regions (Couso and Patraquim, 2017). Although long non-coding RNAs (lncRNAs) are not defined as encoding proteins, some lncRNAs contain sORFs that are engaged by the ribosome, potentially encoding sPeptides (Ruiz-Orera et al., 2018; Ruiz-Orera and Albà, 2019).

sPeptides play important regulatory roles in multiple physiological processes, including growth, development, reproduction, and stress responses (De Coninck and De Smet, 2016). For example, the artificial synthesis and application of the sPeptide hormone insulin were one of the greatest achievements of the twentieth century. Small signaling peptides or peptide hormones in plants, such as cystein-rich peptides, are 5–75 aa long and function as signaling molecules in cell-to-cell communication in defense responses, development, reproduction, and plant–bacteria symbiosis (Marshall et al., 2011; Wang S. et al., 2020). A conserved sORF, *TAS3a*, is associated with the biogenesis of *trans*-acting small-interfering RNAs (tasiRNAs) in *Arabidopsis thaliana*, and a small signaling peptide, *IMMUNE RESPONSE PEPTIDE*, regulates the expression of some defense genes and responds to bacterial or fungal infection in rice (*Oryza sativa*) (Bazin et al., 2017; Wang P. et al., 2020). Multiple sPeptides show high expression in root tissues and a tight association with root growth and absorption. Overexpression of the *C-TERMINALLY ENCODED PEPTIDE1* gene inhibited root growth in *Arabidopsis* (Ohyama et al., 2008). In rice, overexpression of the sPeptide genes, *DROUGHT AND SALT STRESS RESPONSE1*, enhanced drought and stress tolerance, and the knockdown of the sPeptide gene, *OsCADMIUM TOLERANT 3* resulted in a decreased tolerance to aluminum (Xia et al., 2013; Cui et al., 2018).

sORFs and sPeptides have been identified through multiple methods, and their functions have been explored in plants. However, to date, only a few sPeptides have been studied through forward genetic screens because the small size of their encoding sORFs makes them difficult to be targeted by the common mutagenesis methods. Thus, the limited availability of verified sPeptides that can be used as training sets limits the ability of machine learning algorithms to predict sPeptides (Zhou et al., 2013). Further complicating sPeptide identification, many algorithms, such as *de novo* genome annotation, exclude putative proteins of <100 aa in length (Basrai et al., 1997; Claverie, 1997). However, 606,285 potential sORFs (25–250 codons) have been identified in the *A. thaliana* genome (Lease and Walker, 2006).

The bioinformatics and experimental methods have been developed to scan sPeptides encoded by sORFs on a genome-wide level. Bioinformatics approaches based on sequence conservation, functional domains or motifs, gene family clustering, and expression support have been used to search for homologs of known peptides and to predict novel peptides, such as the ROT-FOUR-LIKE/DEVIL (RTFL/DVL) family in *Arabidopsis* (Wen et al., 2004; Guillén et al., 2013; Guo et al., 2015). The transcriptome analysis can reveal the expression of transcripts containing candidate sORFs, but it cannot validate the presence of translational products of these sORFs. The ribosome profiling [also called ribosome sequencing (Ribo-seq)] reveals ribosome footprints by extracting and sequencing RNA that is protected by ribosomes; this can resolve three-nucleotide periodicity, enabling precise definition of translated regions within individual transcripts (Wu et al., 2019). However, several factors introduce contamination in Ribo-seq reads, including structured RNAs and RNAs embedded in protein complexes like rRNA and small nucleolar RNAs (snoRNA), as well as scanning or stalled ribosomes that do not engage in translation (Guttman et al., 2013; Guydosh and Green, 2014; Brar and Weissman, 2015; Archer et al., 2016). Additionally, sPeptides can be directly detected globally using mass spectrometry (MS). For example, an integrated peptidogenomic pipeline using high-throughput MS to probe a customized six-frame translation database was generated and applied to identify non-conventional peptides in maize (*Zea mays*) and *Arabidopsis* (Wang S. et al., 2020). However, since peptides with a bigger size and higher abundance have a better chance of being detected by using MS, the identification of sPeptides solely through MS might miss some of the peptides.

In this study, we collected a large-scale dataset including mRNA sequencing (mRNA-seq), Ribo-seq, and MS data from six tissues (each with two replicates) of maize and performed a *de novo* translatome annotation using the RiboCode software (Xiao et al., 2018). We extracted ORFs of 3–300 nucleotides from the dataset and identified 9,388 sORFs potentially encoding sPeptides. These sORFs showed different Kozak region sequences, higher specificity of translation, and high translational efficiency compared with the canonical protein-coding genes. Furthermore, we searched all sORF sequences in the MS data of the corresponding tissues and verified 2,695 sPeptides. These verified sPeptides clustered perfectly with tissues/replicates, and the verified sPeptides showed higher expression and were longer than the unverified sPeptides. Importantly, the expression in translatome of some annotated sPeptides was positively correlated with that of parental genes, which showed the enrichment of multiple functional gene ontology (GO) terms related to abiotic stress and development. Taken together, the results of this study provide a more comprehensive resource for functional analysis of sPeptides and give helpful information for functional genomics analysis.

## Materials and Methods

### Plant Materials

The seeds of maize (*Z. mays* L.) inbred line B73 were planted in a greenhouse under a temperature and a photoperiod of 30°C for 16 h of light and 25°C for 8 h of darkness. The stem, root, leaf, and whole seedling tissues were collected 14-days after planting, and the ear and tassel tissues were collected in the V12 stage with two biological replicates for each tissue.

### Analysis of RNA-Seq and Ribo-Seq Raw Data

The RNA-seq and Ribo-seq data of these plant samples were collected from study (Zhu et al. 2021). A non-coding RNA data set including rRNA, tRNA, and snoRNA sequences were downloaded from the database Rfam (http://rfam.xfam.org/). After removing the adaptors, the collected data were mapped to this dataset using bowtie2 v2.4.1 with default parameters (Langmead and Salzberg, 2012; Kalvari et al., 2018), and the unmapped reads were kept for downstream analysis. The unaligned mRNA-seq and Ribo-seq reads were mapped to the exon sequences and coding sequences (CDSs) of the B73 reference genome (AGPv4), respectively, using STAR v2.7.3 with default parameters (Schnable et al., 2009; Dobin et al., 2013).

### Identification of sORFs and Calculation of Transcriptional and Translational Abundance

By comparing the alignment results in the BAM format with the B73 reference genome (AGPv4), we identified ORFs in different tissues using the RiboCode software (https://github.com/xryanglab/RiboCode) with default parameters (Xiao et al., 2018). ORFs identified using the RiboCode were classified into seven types as follows:

1. “annotated”: ORFs, overlapping with annotated CDSs, have the same start and stop codon with annotated CDSs.
2. “uORF”: ORFs located upstream of annotated CDSs, not overlapping with annotated CDSs.
3. “dORF”: ORFs located downstream of annotated CDSs, not overlapping with annotated CDSs.
4. “Overlap_uORF”: ORFs located upstream of annotated CDSs and overlapping with annotated CDSs.
5. “Overlap_dORF”: ORFs located downstream of annotated CDSs and overlapping with annotated CDSs.
6. “Internal”: ORFs located internal regions of annotated CDSs, but in a different reading frame.
7. “novel”: ORFs derived from non-coding genes or non-coding transcripts of the coding genes.

The ORFs shorter than 300 nucleotides were considered to be sORFs potentially encoding peptides. Then, the coordinate information of all these sORFs was extracted and merged with the B73 reference genome annotation in the GTF format (https://ftp.ensemblgenomes.org/pub/plants/release-50/gtf/zea_mays/Zea_mays.B73_RefGen_v4.50.gtf.gz) using in-house shell scripts and Stringtie v2.1.4 (parameter: –merge -m 0) (Pertea et al., 2015). The aa sequences of all sORFs were generated by RiboCode and merged together for further analysis.

Using the merged genome annotation, transcriptional and translational abundance was calculated by fragments per kilobase of exon model per million mapped reads (FPKM) using Cufflinks v2.2.1 with parameters: -p 5 -G (Trapnell et al., 2012). Only the unique reads of RNA-seq and Ribo-seq mapped to exons and CDSs were used for the calculation of abundance.

### Protein Preparation and MS

The tissue from maize was grounded using liquid nitrogen and then transferred into a 5-mL centrifuge tube with a suitable volume of lysis buffer [1% TritonX-100, 10 mM dithiothreitol, 1% Protease Inhibitor Cocktail, 50 μM 2,6-diamino-3,5-dithiocyanopyridin, 3 μM trichostatin A, 50 mM *N*-arachidonyl maleimide, and 2 mM ethylenediaminetetraacetic acid (EDTA)]. The mixture was sonicated three times on ice using a high-intensity ultrasonic processor (Scientz), and an equal volume of Tris-saturated phenol (pH 8.0) was added and further vortexed for 5 min. The upper phenol phase was transferred to a new centrifuge tube after centrifugation. Then, at least four volumes of ammonium sulfate-saturated methanol were added to precipitate the proteins at −20°C for at least 6 h. After centrifugation, the precipitate with proteins was collected and washed with ice-cold methanol once, followed by ice-cold acetone three times. The protein was redissolved in 8 M urea, and the concentration was determined using a BCA kit based on the instructions of the manufacturer. After digestion with trypsin, the samples were submitted for the MS detection on the Thermo Scientific Q Exactive platform using a label-free method. The resulting MS data were processed with the Maxquant search engine (v.1.5.2.8). Tandem mass spectra were searched against the aa sequences of sORFs identified by RiboCode in the translatome data (Xiao et al., 2018). The mass tolerance for precursor ions was set as 20 ppm in the first search and 5 ppm in the main search, and the mass tolerance for fragment ions was set as 0.02 Da. The false discovery rate (FDR) was adjusted to <1%, and the minimum score for peptides was set to >40.

The MS data of maize leaves sampled from the 14-day-old seedlings were collected from study (Zhu et al. 2021).

### Calculation of Shannon Entropy and Translation Efficiency (TE)

To compare the TE of sORFs and other canonical transcripts in different tissues, we selected transcripts expressing in both the transcriptome and translatome (FPKM ≥ 0.5). RPKM for single-end sequencing is the unit to quantify gene's expression level, equivalent to FPKM for pair-end sequencing. The RPKM is defined as follows:

$$\begin{array}{l} RPKM=\frac{\text{Exon Mapper Reads }*\;1,000,000,000}{\text{Total Mapped Reads }*\text{ Exon Length}} \end{array}$$

In the pair-end sequencing two paired reads is a fragment. The FPKM is defined as follows:

$$\begin{array}{l} FPKM=\frac{\text{Exon Mapper Fragments }*\;1,000,000,000}{\text{Total Mapped Fragments }*\text{ Exon Length}} \end{array}$$

For each transcript, we calculated TE by the following equation:

$$\begin{array}{l} TE\;=\;\frac{RPKM\;of\;Ribo-seq}{FPKM\;of\;RNA-seq} \end{array}$$

We compared the tissue specificity of expression between verified and non-verified peptides by analyzing the Shannon entropy of peptides. For each sORF, we defined the Shannon entropy of expression-level across different tissues as follows: Given expression levels of a sORF in *N* tissues, the proportion of expression in tissue *i* out of the sum of all expression-levels in all tissues:

$$P(i)\;=\;\frac{FPKM(Ti)}{\sum_{i=1}^{N}FPKM(Ti)}$$

Shannon entropy:

$$SE\;=\;-\sum_{i=1}^{N}P(i)\text{log}2\;[P(i)]$$

### Clustering, Gene Ontology Enrichment Analysis, and Statistical Analysis

The verified and previously annotated 501 peptides were clustered into three groups using the gplots package in R with default parameters. For each group of peptides, the GO enrichment analysis was performed on their parental genes using the AgriGO v 2.0 webserver (Tian et al., 2017). The GO terms with an FDR threshold of 0.05 were considered as significant terms.

All of the statistical analyses in this study were performed using R version 4.0.4.

## Results

### The Widespread Existence of sORFs in Maize

**Figure 1:**
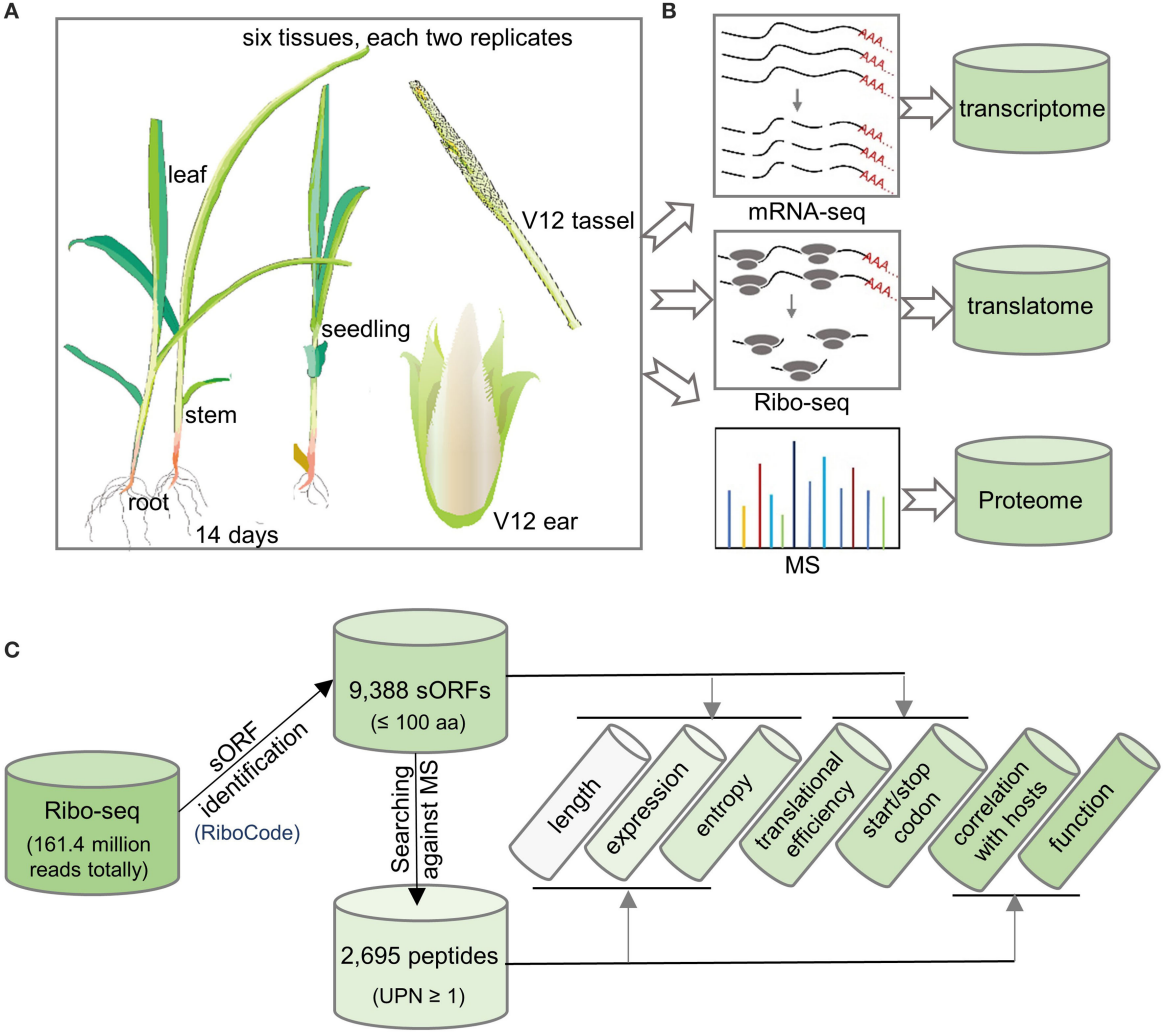
Flowchart of experimentsfor the genome-wide identification of sORFs and sPeptides in maize. **(A)** Six tissues each with two replicates were analyzed in this study. **(B)** Multi-omics data, including transcriptome, translatome, and proteome, were collected for the detection of sORFs and sPeptides. **(C)** Bioinformatics pipeline used in this study to characterize the sORFs and sPeptides.

In a previous study by authors, we sampled whole seedlings, roots, stems, and leaves of 14-day-old maize plants, as well as ears and tassels at the V12 stage with two replicates each and used these to perform RNA sequencing (RNA-seq), Ribo-seq, and MS analysis (MS only for leaf samples) (Figures 1A,B and Supplementary Table 1) (Zhu et al., 2021). We obtained a total of 236 million RNA-seq reads to quantify transcript abundance. We also collected 161.4 million Ribo-seq reads to map ribosome occupancy on genome-wide transcripts (Brar and Weissman, 2015). Additionally, MS was performed to detect and quantify protein abundance for all samples (except the leaf samples) in this study. Taking these results together, we collected a comprehensive transcriptome, translatome, and proteome dataset for the genome-wide identification of sORFs and sPeptides in maize (Figure 1B).

After filtering low-quality reads and contaminant reads in the Ribo-seq data (as shown in the “Materials and Methods” section), we mapped the remaining reads to the maize B73 reference genome (Schnable et al., 2009). By applying stringent filters using the RiboCode software, we identified 9,388 sORFs potentially encoding putative sPeptides (Xiao et al., 2018). Comparison of sORF annotation with the latest maize reference annotation B73 RefGen v4.50 (ensembl) demonstrated 7,589 (80.84%) newly identified sORFs, which complements the functional annotation of the maize genome. Finally, we verified the presence of sPeptides using the MS analysis and identified 2,695 sPeptides, which are supported by the presence of one or more unique peptides (UPN ≥ 1) in the MS analysis (Figure 1C). These results indicate the widespread existence of sORFs and sPeptides in maize.

### Maize sORFs Are a Class of Distinct Translational Elements

**Figure 2:**
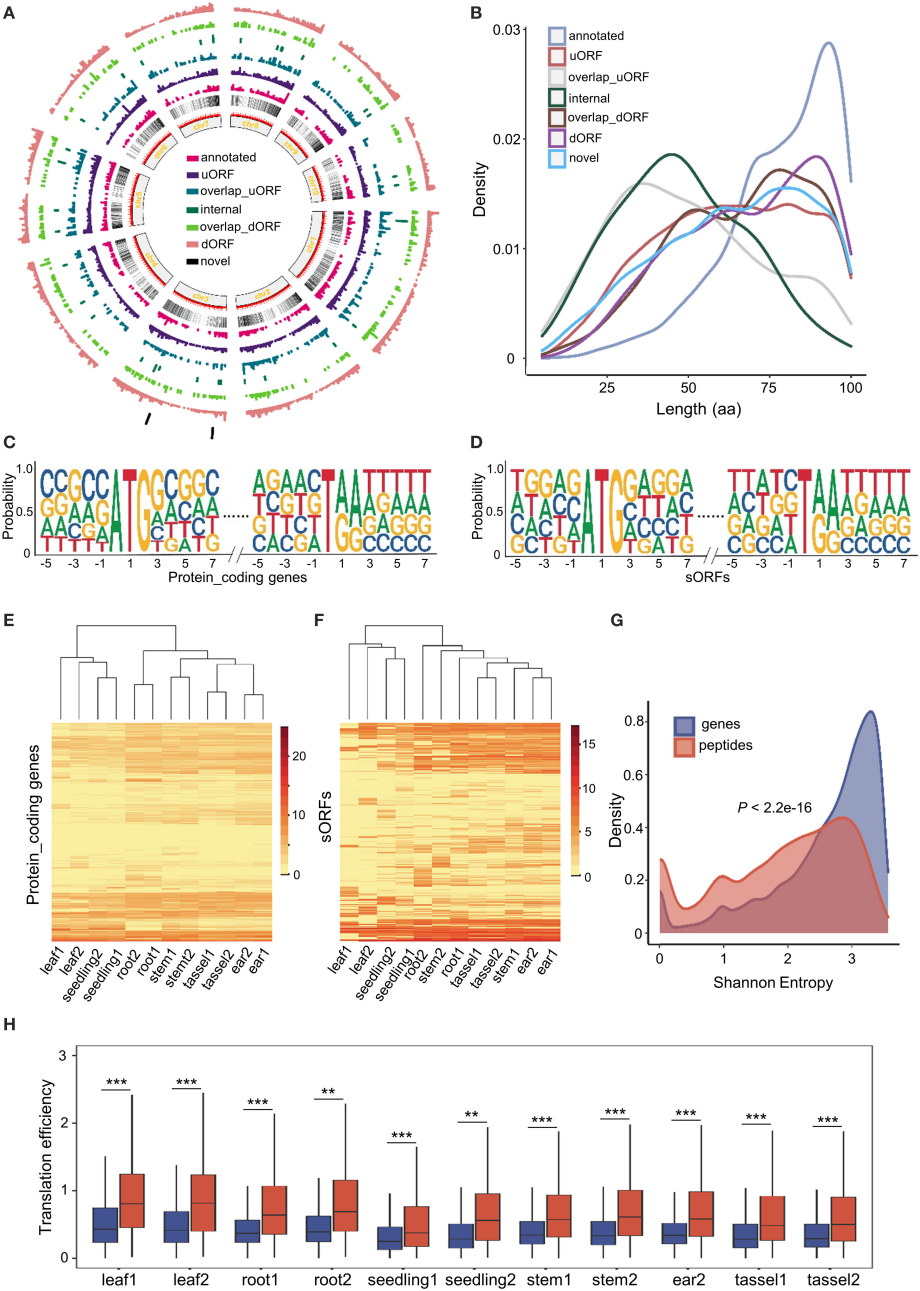
Genome-wide characterization of sORFs in maize. **(A)** Distribution of different kinds of sORFs in the maize genome. **(B)** Length distribution of different kinds of sORFs. **(C,D)** Nucleotide features around start and stop codon sites of conventional genes and sORFs. **(E,F)** Heatmap of expression levels and unsupervised tissue hierarchical trees of conventional genes and sORFs, respectively. **(G)** Shannon entropy distribution of conventional genes (in blue) and sORFs (in red). **(H)** Differentiation of translation efficiency (TE) between conventional genes (mentioned in blue color) and sORFs (mentioned in red color). *,** and *** indicate *P*-values of less than 0.05, 0.01 and 0.001, respectively (Mann–Whitney *U*-test).

By comparing data from this study to the B73 reference genome annotation, we uncovered a total of 2,907 (30.97%) upstream sORFs (uORFs), 485 (5.17%) overlap upstream sORFs (overlap uORFs), 300 (3.2%) internal sORFs, 301 (4.27%) overlap downstream sORFs (overlap dORFs), 3,445 (36.7%) downstream sORFs (dORFs), 1,799 (19.16%) annotated sORFs, and 49 (0.52%) novel sORFs (as shown in the “Materials and methods” section). Similar to genes, sORFs are more likely to be enriched toward the telomeres and depleted in the pericentromeric regions (Figure 2A). All sORFs encoded sPeptides shorter than 100 aa in length. However, the different types of sORFs showed distinct length distribution. Annotated sORFs were significantly longer than other types of sORFs (*p* < 2.2e-16, Mann–Whitney *U* test), while novel and internal sORFs were significantly shorter than the others (*p* < 2.445e-09, *p* < 2.2e-16, respectively, Mann–Whitney *U* test) (Figure 2B). Interestingly, the three shortest uORFs potentially encode peptides with only 5 aa.

The upstream and downstream sequences of start and stop codon sites might be associated with the translational efficiency of ORFs (Hinnebusch et al., 2016). Thus, we extracted the upstream and downstream 5 bp sequences of start and stop codons of the sORFs as well as canonical transcripts and further performed a motif enrichment using R package ggseqlogo (Wagih, 2017). We found that the sequences of the start and stop codons of sORFs were roughly similar to those of conventional genes. However, the frequency of upstream and downstream sequences of start and stop codons was significantly different between sORFs and other canonical transcripts (Figures 2C,D). These results suggest that sORFs exhibit different features (e.g., the frequency of translational start sites) compared with conventional genes.

Moreover, we quantified and compared the expression levels of sORFs and conventional transcripts at the translatome level. Although the translatome abundance of both conventional genes and sORFs could discriminate different tissues, the expression patterns differed dramatically among the different tissues (Figures 2E,F). Furthermore, we calculated the Shannon entropy of expression levels across all 12 samples for sORFs and other protein-coding transcripts and demonstrated that sORFs are significantly more likely to be tissue-specific than the canonical transcripts (*p* < 2.2e-16, Mann–Whitney *U* test) (Figure 2G).

Additionally, we checked the difference in translational efficiency between sORFs and canonical protein-coding genes across all tissues. Unexpectedly, sORFs exhibited significantly higher translational efficiency than canonical protein-coding genes for all 11 samples (the sequencing library of ear1 for RNA sequencing was constructed unsuccessfully) (Figure 2H), which is likely associated with the different base frequency upstream and downstream of the start and stop codons. All these results indicate that sORFs exhibit distinct genomic and expression features compared with canonical protein-coding genes.

### Genome-Wide Classification and Characterization of sPeptides in Maize

**Figure 3:**
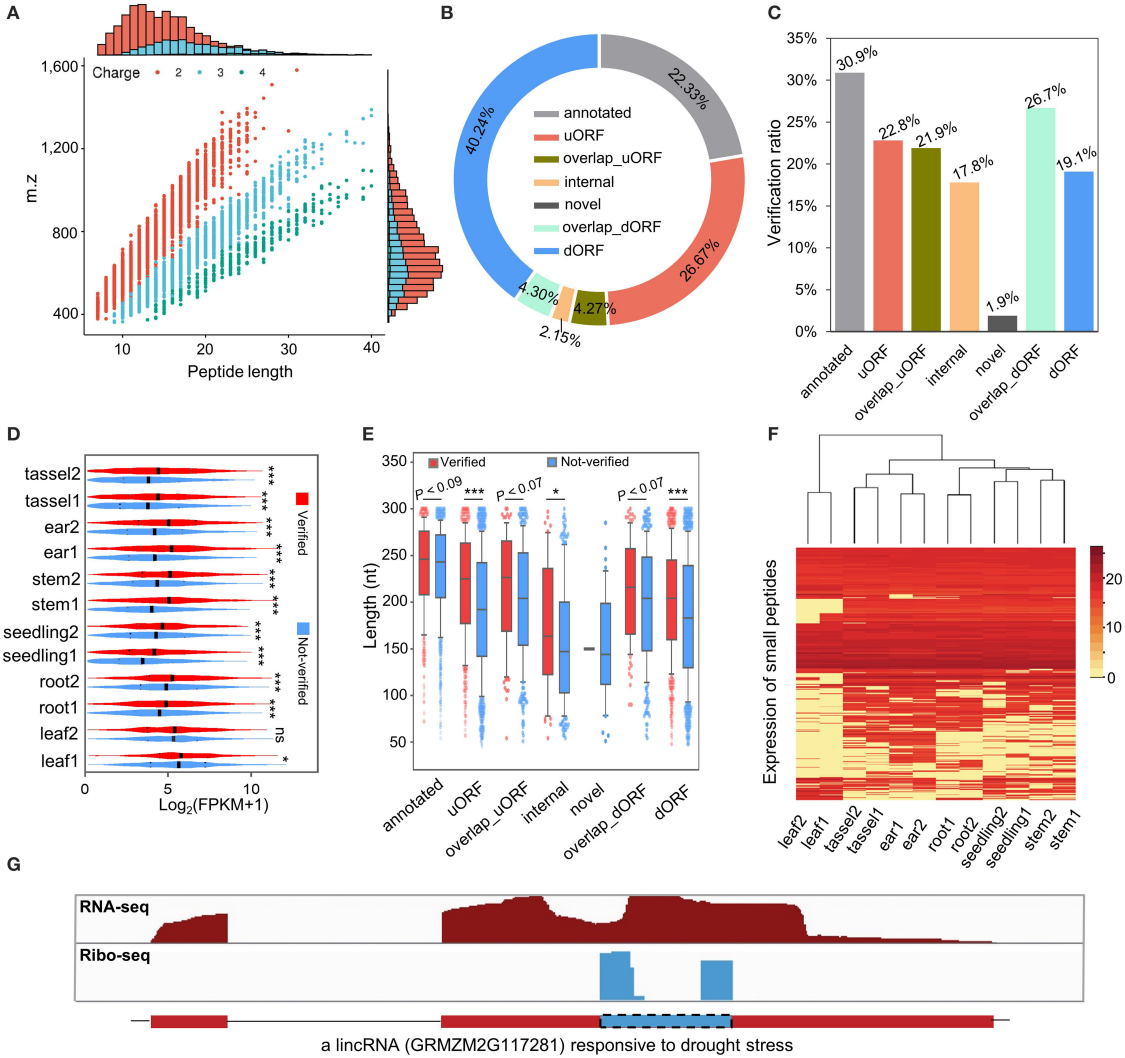
Genome-wide classification and characterization of sPeptides in maize. **(A)** Mass spectrometry (MS) signals discriminate sPeptides and canonical proteins. **(B)** The proportion of different types of sPeptides in maize. **(C)** The validation rate of different kinds of sORFs was detected in this study. **(D)** Comparison of expression abundance between sPeptides and unverified sORFs. *, ** and *** indicate *P*-values of less than 0.05, 0.01 and 0.001, respectively (Mann–Whitney *U*-test). **(E)** Length differentiation between sPeptides and unverified sORFs in maize. *,** and *** indicate *P*-values of less than 0.05, 0.01 and 0.001, respectively (Mann–Whitney *U*-test). **(F)** Expression pattern of sPeptides in the 12 samples. **(G)** Structure of a sPeptide derived from a lincRNA.

To evaluate whether sORFs are able to translate stable peptides in maize, we performed a proteogenomic analysis by searching the aa sequences of sORFs against the MS data (Walley and Briggs, 2015). Consistent with the definition of sPeptides as <100 aa, the sPeptides were mainly concentrated in the range of 0–10 kDa in the MS validation, which corresponds to the length of 5–100 aa (Figure 3A and Supplementary Figure 1A). By using the MS analysis, we verified 2,596 sPeptides of the 9,388 sORFs identified from Ribo-seq data. Of these sPeptides, ~85% were detected in more than five tissues (Supplementary Figure 1B). Unexpectedly, only about 20% of sPeptides were derived from annotated transcripts of the reference genome, suggesting that the number of sPeptides in maize is largely underestimated (Figure 3B). However, the comparison of the validation ratio of sPeptides derived from different types of sORFs demonstrated that sORFs annotated previously in the reference genome are most likely to be validated, while those from novel sORFs are least likely to be validated (Figure 3C).

The comparison of Shannon entropy between different kinds of sPeptides and unverified sORFs indicated that sPeptides with MS evidence were more uniformly expressed in the 12 samples (Supplementary Figure 1C). Notably, sPeptides exhibit higher translatome abundance and longer CDS in corresponding sORFs than those of the unverified sORFs, which may be due to the limitation of the detection power of MS (Figures 3D,E). Then, we clustered the 12 samples based on the expression-level variation of all detectable sPeptides and found that the sPeptides can robustly discriminate tissues, and the replicates of the same tissue were also clustered (Figure 3F). Interestingly, we found that many sPeptides are derived from putative-defined lncRNA regions. For example, a long intergenic non-coding RNA (lincRNA) *GRMZM2G117281_T01* annotated in the reference genome of maize was detected to be translated to a sPeptide (Figure 3G). A previous study proposed that *GRMZM2G117281* could be responsive to drought stress (Zhang et al., 2014), suggesting a potential functional role of this sPeptide. All these results demonstrate that sPeptides are abundant in the maize genome, exhibit distinct genomic features, and may function in different biological processes.

### Annotated sPeptides Are Correlated With Their Corresponding Parental Genes and Might Function in Multiple Processes

**Figure 4:**
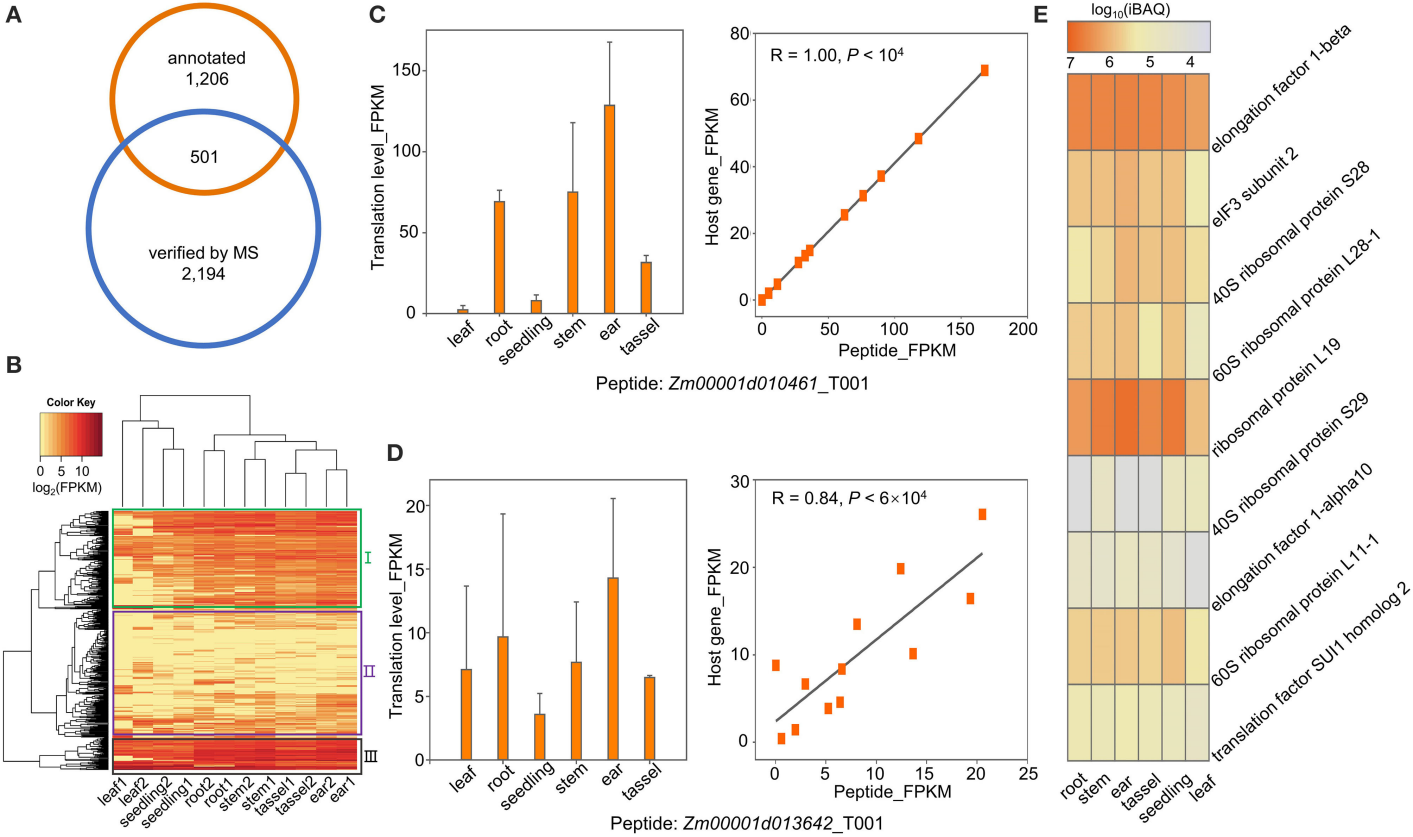
Functional annotation of sPeptides in maize. **(A)** Gene-derived sPeptides were identified by the intersection between annotated genes and sPeptides in this study. **(B)** Gene-derived sPeptides classified different tissues and clustered them into three groups based on their expression patterns. **(C,D)** Translatome abundance variation of sPeptides Zm00001d010461_T001 **(C)** and Zm00001d013642_T001 **(D)** across different tissues shows a high correlation with that of their parental canonical protein-coding gene. **(E)** Proteome abundance of sPeptides across different tissues from Group III.

A fraction of the ORFs located within annotated genes was shorter than 300 nucleotides. Some of them have been demonstrated to be capable of encoding sPeptides with biological functions such as signal peptides (Hsu and Benfey, 2018; Ruiz-Orera and Albà, 2019). In this study, we compared the sPeptides with the functional annotation in the maize B73 RefGen v4 reference genome. A total of 501 out of 2,596 sPeptides identified by Ribo-seq and MS were encoded by sORFs annotated in the reference genome (Figure 4A). Based on the expression of the sPeptides quantified by Ribo-seq, we clustered 501 genes into three groups (Figure 4B). Furthermore, the translatomic abundance of 501 sPeptides was positively correlated with that of their host genes (Supplementary Figure 2D), suggesting that sPeptides might function by orchestrating parental gene expression.

To explore the potential functions of sPeptides, we performed the GO enrichment analysis for the three parental gene sets that correspond to the three different sPeptide groups. Group I parental genes were significantly enriched in the RNA splicing process (Supplementary Figure 2A). *Zm00001d010461, Zm00001d042725*, and *Zm00001d024593*, enriched in the GO term “RNA splicing,” are annotated to encode small nuclear ribonucleoprotein family-like (LSM) proteins. Generally, LSM proteins are associated with development, response to stress, and abscisic acid signaling *via* mRNA splicing and processing in *Arabidopsis* (Xiong et al., 2001; Perea-Resa et al., 2012; Golisz et al., 2013; Cui et al., 2014; Okamoto et al., 2016). The abundance of the sPeptide, Zm00001d010461_T001 varied dramatically in different tissues and showed a highly positive correlation with the expression of *Zm00001d010461* (*R* = 1, *p* < 1e-04) (Figure 4C). Therefore, we speculated that the sPeptide, Zm00001d010461_T001 could function in stress tolerance and development in maize.

For the second group of parental genes, the GO enrichment showed that these genes are related to organonitrogen compound biosynthetic and metabolic processing (Supplementary Figure 2B). Nitrogen is essential for plant growth and development and is also helpful for tolerance against biotic and abiotic stresses (Oh et al., 2017; Zipfel and Oldroyd, 2017; Arora et al., 2020). Notably, *Zm00001d034602* is annotated to function in the organonitrogen compound metabolic process, and its abundance in the translatome was significantly associated with that of the sPeptide Zm00001d034602_T001 (Figure 4D). *AT2G22425*, the ortholog of *Zm00001d013642* in *Arabidopsis*, was reported to function in signal peptide processing during *Cabbage leaf curl virus* infection, indicating that the sPeptide Zm00001d013642_T001 may be involved in pathogen response through organonitrogen compound biosynthetic and metabolic processing (Ascencio-Ibáñez et al., 2008).

The sPeptide parental genes in Group III were uniformly expressed across different tissues and showed high abundance at both the translatome and proteome levels (Figures 4B,E). These genes are mainly enriched in translation and peptide biosynthetic processes (Supplementary Figure 2C). Nine genes with the most significant GO enrichment of translation are annotated as “subunit of ribosome and translation-regulatory factors.” These peptides are likely to participate in the fundamental biological pathways and affect multiple agronomic traits.

## Discussion

As a new frontier in the study of molecular players in life science, sORFs and sPeptides have been reported to be involved in several biological processes in plants. In this study, we collected RNA-seq, Ribo-seq, and MS data from six tissues of the maize reference inbred B73 with two replicates and performed a genome-wide *de novo* scan of translational elements using the RiboCode software (Xiao et al., 2018). We extracted ORFs encoding peptides with length ranging from 2 to 100 aa and identified 9,388 sORFs potentially encoding sPeptides. Then, we confirmed over 2,000 sPeptides by searching against MS data. Comparison of sORFs/sPeptides with canonical proteins demonstrated the distinct features of these non-canonical molecules. Finally, the functional GO analyses indicated that these sPeptides are likely to be involved in the response to abiotic stress and plant development (Couso and Patraquim, 2017). This study presents a biological pipeline combining multi-omics data for the genome-wide scan of sORFs and sPeptides in maize, which paves the way for further functional study of sPeptides in maize.

### A Robust Bioinformatics Pipeline for the Genome-Wide Scan sORFs and sPeptides Combining Multi-Omics Data of RNA-Seq, Ribo-Seq, and MS

lncRNAs have a low probability of encoding proteins and are more likely to encode sPeptides. lncRNAs have been widely uncovered by RNA-seq in maize (Li et al., 2014). The sORFs or sPeptides are detected by the bioinformatics analyses solely based on RNA-seq data lack representation at the translatome level. Ribosome profiling (Ribo-seq), an RNA sequencing technique focused on the translatome, has been developed and used to monitor translation in real-time during protein biosynthesis (Ingolia et al., 2009). Since ribosomes directly decipher mRNA every three nucleotides, the periodic feature of ribosome footprints can be used to examine unannotated ORFs (Calviello et al., 2016; Hsu et al., 2016). Therefore, sORFs potentially encoding peptides that may not be detected by proteomics-based methods could be uncovered by combining ribosome profiling and bioinformatics analyses (Couso and Patraquim, 2017; Olexiouk et al., 2018; Ruiz-Orera and Albà, 2019).

Due to the presence of reads from the elongating and initiating stage in Ribo-seq experiments, non-AUG start site prediction becomes difficult and false-positive results are introduced (Olexiouk et al., 2018). Wang et al. generated an integrated proteomic pipeline using high-throughput MS to probe a customized six-frame translation database and applied it to a large-scale detection of non-conventional peptides in plants (Wang S. et al., 2020). In total, 1,993 and 1,860 non-conventional peptides were identified in maize and *Arabidopsis*, respectively. However, limited by the systematic error of the technology, in which proteins with higher abundance or larger size have better chances of being detected by the MS, the approaches used to identify peptides based on their mass spectra are likely to have high rates of false negatives (Slavoff et al., 2013; Olexiouk et al., 2018).

Thus, we generated a pipeline for the genome-wide scan of sORFs and sPeptides in maize combining sensitive and real-time Ribo-seq monitoring data as well as less-sensitive but direct MS data. In this study, the annotated sORFs had the highest verification ratio (Figure 3C), which may be associated with the fact that annotated sORFs generally have longer length than the other types of sORFs (Figure 2B). The corresponding peptides of annotated sORFs are more easily identified by the MS because of their larger molecular mass. We confirmed 2,695 sPeptides by the MS, which accounted for 28.71% of all identified sORFs (9,388). Therefore, Ribo-seq provides an unprecedented chance to detect more potential sPeptides likely with low abundance or smaller relative molecular mass that are difficult to detect by the proteomics-based methods.

To further validate the capacity of pipeline of authors to detect sPeptides, we downloaded and analyzed the MS data from the previous study which identified non-conventional peptides in maize-based on a peptidogenomic method (Wang S. et al., 2020). All sORFs identified from the Ribo-seq data of six different maize tissues were searched against the downloaded MS data that were obtained only from maize leaves at the V3 stage, and 158 sORFs showed peptides evidence. Of these validated sORFs by Wang's MS data, 66 sORFs were also verified in the total MS data (Supplementary Figure 3). Although the number and types of tissues are different, there is a proportion of overlapped sPeptides (~42%) between this study and the previous study based on the non-digestion MS data, reflecting a certain degree of reliability in pipeline of authors. Moreover, the sPeptides, identified in this study, are complementary to previous studies to extend the knowledge of sPeptides in maize.

### Potential Functional Roles of sPeptides

Numerous studies have reported that small secreted peptides are involved in different physiological processes including plant growth, development, reproduction, and stress responses (De Coninck and De Smet, 2016). Cysteine-rich peptides, a type of signal peptide, play important roles in developmental patterning as well as in plant–pathogen responses and symbiosis (Hemu et al., 2018). Based on a proteomics method, 1,993 unannotated peptides were identified in maize leaves, which were significantly enriched in regions identified from genome-wide association studies of agronomic traits and appear to be under domestication selection (Wang S. et al., 2020). Thus, the sPeptides identified in this study are likely to function in multiple pathways in maize.

Based on the expression patterns between tissues, the 501 gene-locus-encoded sPeptides annotated in the reference genome and verified by the MS were clustered into three groups through a hierarchical clustering algorithm (Figure 4B). Genes in Group I were significantly enriched in the pathway related to mRNA splicing and processing. A large number of studies reported that alternative splicing plays vital roles in growth, development, and responses to stress (de Francisco Amorim et al., 2018; Szakonyi and Duque, 2018; Li et al., 2021). In this study, the sPeptide-associated parental genes, *Zm00001d010461, Zm00001d042725*, and *Zm00001d024593*, which are annotated as LSM protein family genes, belong to Group I. In *Arabidopsis*, LSM proteins participate in mRNA splicing and degradation and thus regulate the development and tolerance to stress (Perea-Resa et al., 2012; Golisz et al., 2013; Cui et al., 2014; Okamoto et al., 2016). Moreover, *SAD1*, encoding a polypeptide similar to multifunctional LSM proteins, modulates abscisic acid signal transduction and biosynthesis in *Arabidopsis* through mRNA metabolism (Xiong et al., 2001). The second group (Group II) of sPeptide parental genes was enriched in organonitrogen compound biosynthetic and metabolic processes (Supplementary Figure 2B). Signal molecules, such as reactive oxygen species, calcium, reactive nitrogen species, salicylic acid, and ethylene, are important in plant development and pathogen infection (Oh et al., 2017; Zipfel and Oldroyd, 2017). The form of nitrogen, such as nitrate or ammonium, plays a vital role in the production of these signal molecules (Oh et al., 2017; Arora et al., 2020). The parental genes of the Group II sPeptide, *Zm00001d013642_T001* and *Zm00001d013642*, were annotated in “organonitrogen compound metabolic processing,” and its ortholog *AT2G22425* was differentially expressed in *Cabbage leaf curl virus*-infected *Arabidopsis* leaves (Ascencio-Ibáñez et al., 2008). These results imply that *Zm00001d013642_T001* may be involved in signal transduction during the pathogen response through organonitrogen compound biosynthetic and metabolic processes. The last group (Group III) of genes were uniformly expressed in different tissues and with relatively high abundance at the translatome level (Figure 4B). There were nine sPeptide parental genes annotated as “subunit of translation start and elongation factors” in Group III. Overall, sPeptides might participate in multiple pathways in different manners to influence the plant life cycle.

### Precursors of miRNAs May Encode sPeptides

Some lncRNAs and circular RNAs were detected *in vivo* with evidence for the production of peptides in the shotgun MS data (van Heesch et al., 2019), which implies that non-coding transcripts are likely to play both coding and non-coding roles. A previous study reported that microRNAs (miRNAs) are capable of encoding peptides through the transcripts of their corresponding precursors (Chen et al., 2020). Primary miRNAs have been reported to encode regulatory peptides in *Arabidopsis*, grapevine (*Vitis vinifera*), soybean (*Glycine max*), and *Medicago* sp.; these peptides are named miRNA-encoded peptides (miPEPs) (Ren et al., 2021). For example, miPEP171d1 plays a regulatory role in adventitious root formation and response to stress in plants (Ma et al., 2014; Gao et al., 2019; Chen et al., 2020).

In this study, we aligned the aa sequences of identified sPeptides against the reference sequences of the precursors of miRNAs using BLAST+ (version 2.7.1). Two sPeptides validated by the MS are encoded by pre-miRNAs: ENSRNA049997513-T1 and ENSRNA049997089-T1. In a previous study, zma-miR159d, the mature product of ENSRNA049997513-T1, was predicted to target genes encoding MYB transcription factors (Samad et al., 2017). Moreover, zma-miR159d was found to be involved in the degradation of chlorophyll that induced earlier leaf senescence between different maize inbred lines (Wu et al., 2016). Considering the evidence of their translatome and proteome study, we speculated that zma-miR159d could produce a sPeptide. The other sPeptide is encoded by ENSRNA049997089-T1, which is the primary transcript of *zma-MIR2275d*. *zma-MIR2275*, with a maximum expression in the fertile maize anther, plays an important role in anther development and thereby influencing male reproduction in maize (Zhai et al., 2015; Huang et al., 2019). Furthermore, zma-miR2275 also affects the drought tolerance by directly targeting drought-related mRNAs. The identification of the sPeptide encoded by the pre-miRNA ENSRNA049997089-T1 provides a perspective on the manner of *zma-MIR2275* function as a regulator of development and stress tolerance in maize.

## Data Availability Statement

The MS proteomics data for this project have been deposited at the ProteomeXchange Consortium with the dataset identifier PXD025997.

## Author Contributions

LL designed and supervised this study. YL, WZ, SC, and JQ performed the data analysis. YL, WZ, and LL prepared the manuscript. All authors contributed to the article and approved the submitted version.

## Conflict of Interest

The authors declare that the research was conducted in the absence of any commercial or financial relationships that could be construed as a potential conflict of interest.

## Supplementary Material

Supplementary Figure 1 Size distribution of peptides in MS and comparison between verified and unverified sORFs. **(A)** Statistics of the mass of peptides. **(B)** Percentage of verified sORFs and their composition. **(C)** Comparison of Shannon entropy between different kinds of verified and unverified sORFs.

Supplementary Figure 2 The results of GO enrichment analysis of three groups of sPeptide-associated parental genes and correlation of abundance between the 501 sPeptides and their parental genes. **(A–C)** Results of GO enrichment analysis of three clusters of parental genes, respectively. **(D)** The 501 sPeptides positively correlated with their parental genes in the translatome.

Supplementary Figure 3 The overlapped sPeptides validated by MS data of this study and Wang's study.

Supplementary Table 1 Tissues used in this study.

Supplementary File 1 Genome annotation of sPeptides in GTF format.

Supplementary File 2 Information of sORFs and sPeptides.
