# Supplementary figures and images for: Genome-Wide Identification and Characterization of Small Peptides in Maize
Source: Front Plant Sci. 2021 Jun 16;12:695439. doi: 10.3389/fpls.2021.695439 (PMC8244733; doi:10.3389/fpls.2021.695439)

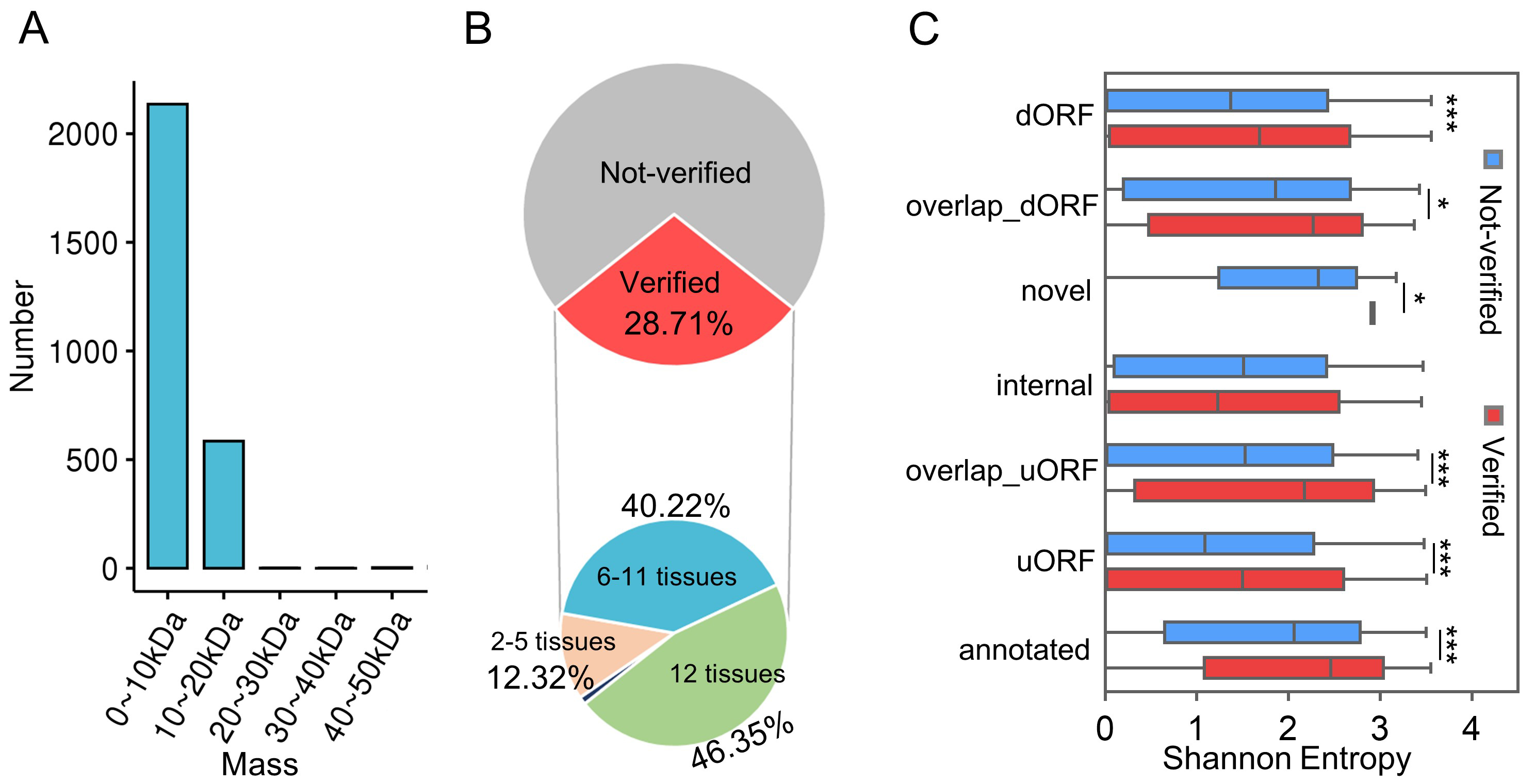

Supplement: Supplementary Figure 1 — Size distribution of peptides in MS and comparison between verified and unverified sORFs. (A) Statistics of the mass of peptides. (B) Percentage of verified sORFs and their composition. (C) Comparison of Shannon entropy between different kinds of verified and unverified sORFs. [file Image_1.JPEG]

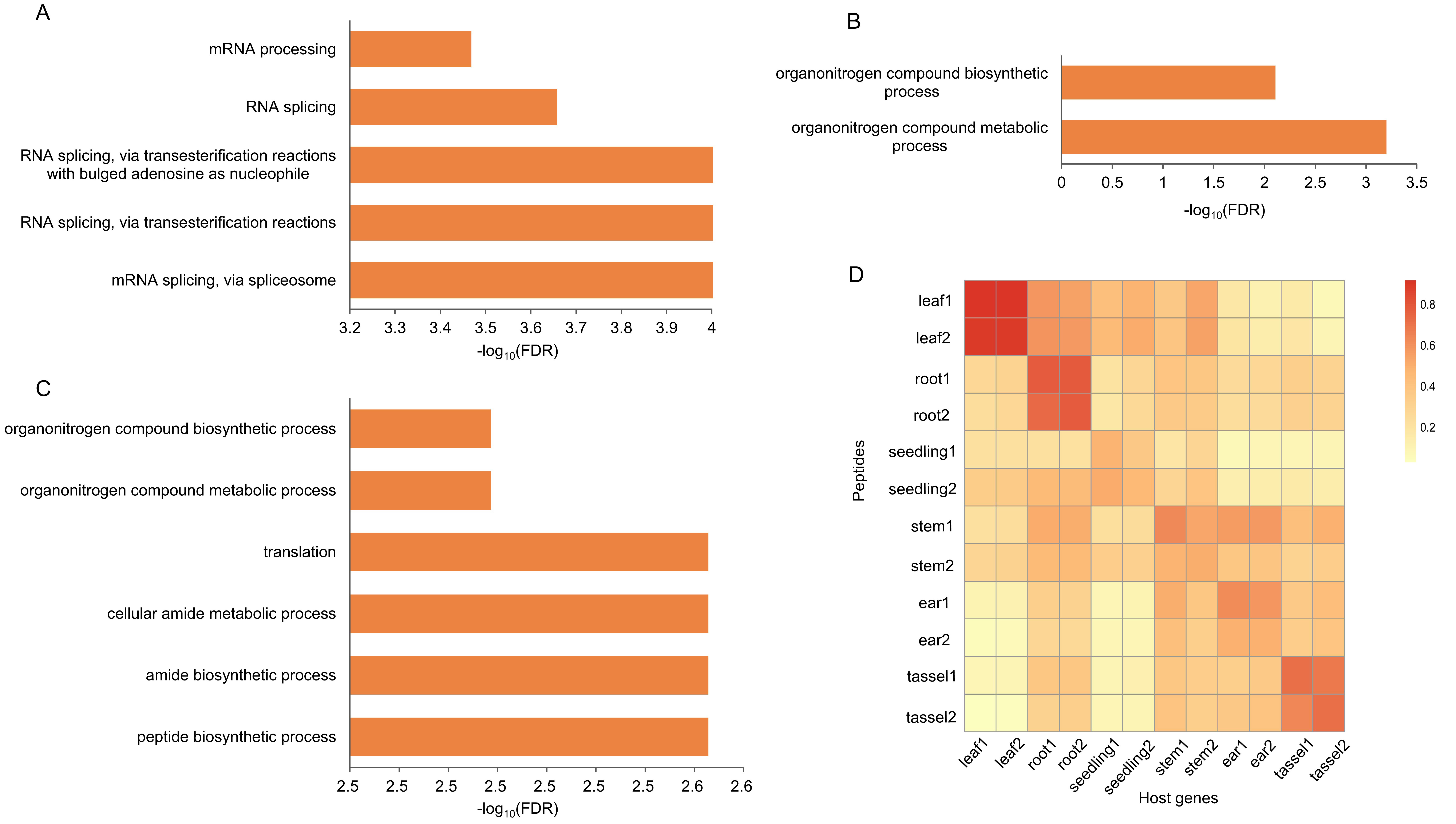

Supplement: Supplementary Figure 2 — The results of GO enrichment analysis of three groups of sPeptide-associated parental genes and correlation of abundance between the 501 sPeptides and their parental genes. (A–C) Results of GO enrichment analysis of three clusters of parental genes, respectively. (D) The 501 sPeptides positively correlated with their parental genes in the translatome. [file Image_2.JPEG]

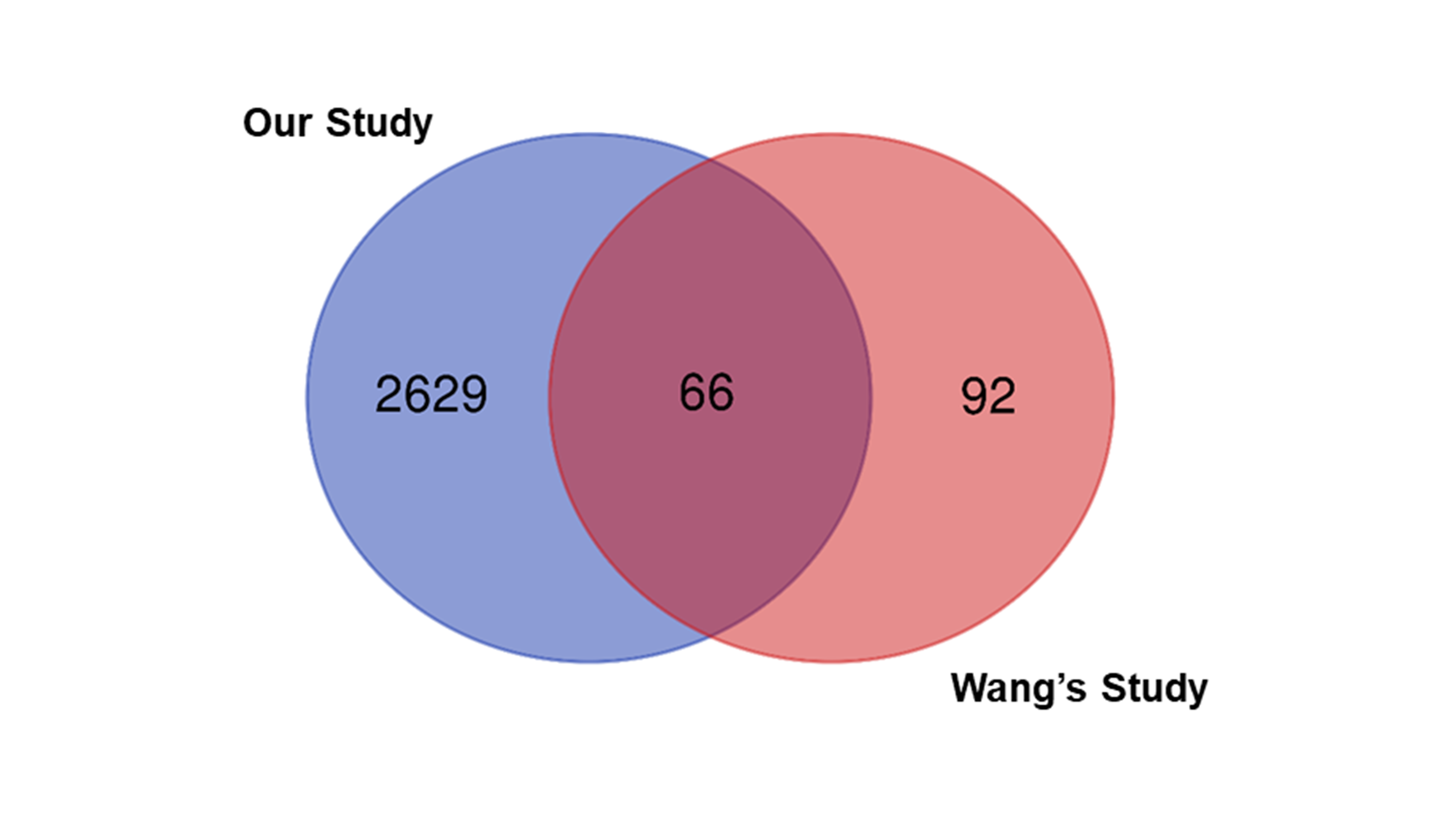

Supplement: Supplementary Figure 3 — The overlapped sPeptides validated by MS data of this study and Wang's study. [file Image_3.TIF]
