# Supplementary material for: Genome-Wide Identification and Characterization of Small Peptides in Maize
Source: Front Plant Sci. 2021 Jun 16;12:695439. doi: 10.3389/fpls.2021.695439 (PMC8244733; doi:10.3389/fpls.2021.695439)
Supplement: Supplementary Table 1 — Tissues used in this study. [file Table_1.DOCX]

| Tissue | Data | ref |
| --- | --- | --- |
| 14 days seedling | RNA-seq | Zhu et al., 2021 |
| 14 days seedling | RNA-seq | Zhu et al., 2021 |
| 14 days leaf | RNA-seq | Zhu et al., 2021 |
| 14 days leaf | RNA-seq | Zhu et al., 2021 |
| 14 days root | RNA-seq | Zhu et al., 2021 |
| 14 days root | RNA-seq | Zhu et al., 2021 |
| 14 days stem | RNA-seq | Zhu et al., 2021 |
| 14 days stem | RNA-seq | Zhu et al., 2021 |
| V12 stage ear | RNA-seq | Zhu et al., 2021 |
| V12 stage tassel | RNA-seq | Zhu et al., 2021 |
| V12 stage tassel | RNA-seq | Zhu et al., 2021 |
| 14 days seedling | Ribo-seq | Zhu et al., 2021 |
| 14 days seedling | Ribo-seq | Zhu et al., 2021 |
| 14 days leaf | Ribo-seq | Zhu et al., 2021 |
| 14 days leaf | Ribo-seq | Zhu et al., 2021 |
| 14 days root | Ribo-seq | Zhu et al., 2021 |
| 14 days root | Ribo-seq | Zhu et al., 2021 |
| 14 days stem | Ribo-seq | Zhu et al., 2021 |
| 14 days stem | Ribo-seq | Zhu et al., 2021 |
| V12 stage ear | Ribo-seq | Zhu et al., 2021 |
| V12 stage ear | Ribo-seq | Zhu et al., 2021 |
| V12 stage tassel | Ribo-seq | Zhu et al., 2021 |
| V12 stage tassel | Ribo-seq | Zhu et al., 2021 |
| 14 days seedling | MS | - |
| 14 days seedling | MS | - |
| 14 days leaf | MS | Zhu et al., 2021 |
| 14 days leaf | MS | Zhu et al., 2021 |
| 14 days root | MS | - |
| 14 days root | MS | - |
| 14 days stem | MS | - |
| 14 days stem | MS | - |
| V12 stage ear | MS | - |
| V12 stage ear | MS | - |
| V12 stage tassel | MS | - |
| V12 stage tassel | MS | - |
